# Nano-Wilhelmy investigation of dynamic wetting properties of AFM tips through tip-nanobubble interaction

**DOI:** 10.1038/srep30021

**Published:** 2016-07-25

**Authors:** Yuliang Wang, Huimin Wang, Shusheng Bi, Bin Guo

**Affiliations:** 1 Robotics Institute, School of Mechanical Engineering and Automation, Beihang University, Beijing 100191, P.R. China; 2 Department of Materials Science and Engineering, The Ohio State University, 2041 College Rd., Columbus, OH 43210, USA; 3 School of Material Science and Engineering, Harbin Institute of Technology, Harbin, 150001, P.R. China

## Abstract

The dynamic wetting properties of atomic force microscopy (AFM) tips are of much concern in many AFM-related measurement, fabrication, and manipulation applications. In this study, the wetting properties of silicon and silicon nitride AFM tips are investigated through dynamic contact angle measurement using a nano-Wilhelmy balance based method. This is done by capillary force measurement during extension and retraction motion of AFM tips relative to interfacial nanobubbles. The working principle of the proposed method and mathematic models for dynamic contact angle measurement are presented. Geometric models of AFM tips were constructed using scanning electronic microscopy (SEM) images taken from different view directions. The detailed process of tip-nanobubble interaction was investigated using force-distance curves of AFM on nanobubbles. Several parameters including nanobubble height, adhesion and capillary force between tip and nanobubbles are extracted. The variation of these parameters was studied over nanobubble surfaces. The dynamic contact angles of the AFM tips were calculated from the capillary force measurements. The proposed method provides direct measurement of dynamic contact angles for AFM tips and can also be taken as a general approach for nanoscale dynamic wetting property investigation.

Atomic force microscopy (AFM)^1^ has been extensively applied in numerous applications because it can achieve high spatial resolution and high force sensitivity^2^,^3^. The wetting properties of AFM probe tips are of concern in AFM tip related force measurement, fabrication, and manipulation techniques, such as “dip-pen” nanolithography^4^, nano-dispensing^5^, micro/nanomanipulation^6^,^7^, and nanotribological investigation^8^, and even basic imaging. In “dip-pen” nanolithography and nano-dispensing, meniscus bridges form between AFM tips and substrates. The wetting properties of AFM tips determine the size and shape of the meniscus bridges, which in turn determine the lithography resolution and transport rate from AFM tips to substrates. In AFM-based micro/nano-manipulation, tribological investigation and imaging, the meniscus force often dominates the adhesion force. As a result, the wetting properties of cantilever tips play crucial roles in these operations and may introduce artifacts into the measurement results^9^.

Micro/nanoscale wetting property measurement techniques can be briefly classified into two groups: force-based methods and imaging-based methods. In force-based methods, fibers or needles are immersed or withdrawn from liquid. The wetting properties can be obtained by measuring corresponding capillary forces exerted on them and employing the Wilhelmy balance method with a known solid-liquid-gas (three phase) contact line. This method has been used to study the wetting properties of carbon nanotubes^10^,^11^ and other fibers with constant diameters^11^,^12^,^13^,^14^. The method typically requires dedicated fabrication to probes to guarantee the constant diameter of the probes.

In imaging-based methods, the wetting properties are obtained by imaging micro/nano-droplets or menisci through scanning electron microscopy (SEM)^15^, transmission electron microscopy (TEM)^16^,^17^, or AFM^18^,^19^. The imaging-based methods are complicated by evaporation of the droplets during measurement^13^,^19^. Additionally, the method is restricted to measurement of static contact angles and cannot be used to measure dynamic contact angles.

Unlike carbon nanotubes which have constant diameters, AFM tips are often pyramid-shaped. To facilitate the modeling of tip-sample interaction, AFM tip geometries are generally simplified as spheres^20^,^21^, truncated cones^22^, or cylinders^23^,^24^. The simplification provides qualitative analysis of tip-sample interaction and does not allow for precise measurement. Other studies have modified the regular cantilevers through focused ion beam (FIB) milling to achieve probe tips with nearly constant diameters.

Due to their geometrical complexity and extremely small size, the wetting properties of AFM tips are usually obtained by indirectly measuring the contact angles on a flat surface of identical material using the sessile drop method^25^. The only direct contact angle measurement of AFM tips was conducted by Tao and Bhushan^26^. In their study, the contact angles were obtained by measuring cantilever deflection changes at snap-in or snap-out points during immersion or withdrawal of the AFM tips. The accuracy of this method is directly related to measurement of the radius of curvature of AFM tips. In the Tao and Bhushan study, the radius of curvature is obtained through “blind tip reconstruction” from scanned images of grating samples, which may introduce errors. Moreover, the calculation of the contact angle in their study is based on the adhesion of a sphere in contact with liquid surfaces. The actual geometry of AFM tip-liquid surface contact is complex and involves a three phase contact line which may be pinned at a pyramidal part of the tip^12^. Additionally, the measurement results of advancing contact angles were not repeatable. As a result, the method was only applicable for the receding contact angle measurement.

In this study, a novel method is proposed to directly measure wetting properties of regular AFM tips at the nanoscale based on the micro-Wilhelmy balance method. In this method, the dynamic contact angles of AFM tips are obtained through capillary force measurement during immersion and retraction motion of the AFM tips relative to interfacial nanobubbles on hydrophobic surfaces. Spherical cap bubbles on various hydrophobic surfaces in aqueous solvents have been widely studied over the last ten years^27^,^28^,^29^,^30^,^31^. These gas bubbles, having heights between 5 and 100 nm and diameters between 100 and 800 nm, are normally referred to as interfacial nanobubbles and have been experimentally confirmed using AFM^24^,^27^,^29^,^31^,^32^ as well as other methods^33^,^34^,^35^,^36^,^37^. In this method, nanobubbles are used to provide stable gas-liquid interfaces in order to employ the nano-Wilhelmy balance method.

Two things need to be done to employ the nano-Wilhelmy balance method to AFM tips. The first is accurate measurement of capillary force during tip-nanobubbe interaction, which can be directly obtained with an AFM. The second is the accurate measurement of the three phase contact lines during measurement. Instead of taking AFM tips as truncated cones or cylinders, we constructed tip geometries from SEM images of the AFM tips taken from different directions. With the constructed tip geometries, the variation of the three phase contact line with respect to immersion depth can be precisely determined.

AFM cantilevers were immersed into water during measurement. This gives several advantages. First, immersion allows for complete force distance curves, from which the conversion factor of cantilever deflection signal can be accurately obtained. A second benefit is in signal correction. It is known that due to optical interference, the cantilever deflection signal is not flat with respect to the motion of AFM scanners^38^. Since the cantilevers are immersed, the signal of cantilever deflection without tip-nanobubble interaction can be taken as a reference signal to correct that obtained during tip-nanobubble interaction. Finally, the liquid-gas interface provided by nanobubbles is stable. This eliminates the fluctuation of liquid-gas interfaces and evaporation that is experienced when performing measurements using liquid droplets or bulk liquid-gas interfaces. To our best knowledge, it is the first time the interfacial nanobubbles were applied to provide stable liquid-gas interfaces in wetting property measurement at the nanoscale.

In this study, the principle of the proposed method was first presented. A mathematic model utilized for the calculation of dynamic contact angles was proposed. Then interfacial nanobubbles were obtained on hydrophobic surfaces. Nanobubble coalescence was conducted to obtain nanobubbles large enough to facilitate dynamic contact angle measurement. The force volume function was applied to the obtained nanobubbles. With the force volume function, force distance curves over the nanobubbles were obtained. The dynamic contact angles of AFM tips were obtained from the force-distance curves.

## Results and Discussion

In this section, the nanobubble imaging and coalescence are first introduced on the PS surface in water. Then the detailed process of tip-nanobubble interaction at different stages is presented. With the force distance curves, the dynamic contact angles are finally obtained and the change of several other parameters over nanobubble surface is investigated.

### Formation of nanobubbles with larger sizes

**Figure 1:**
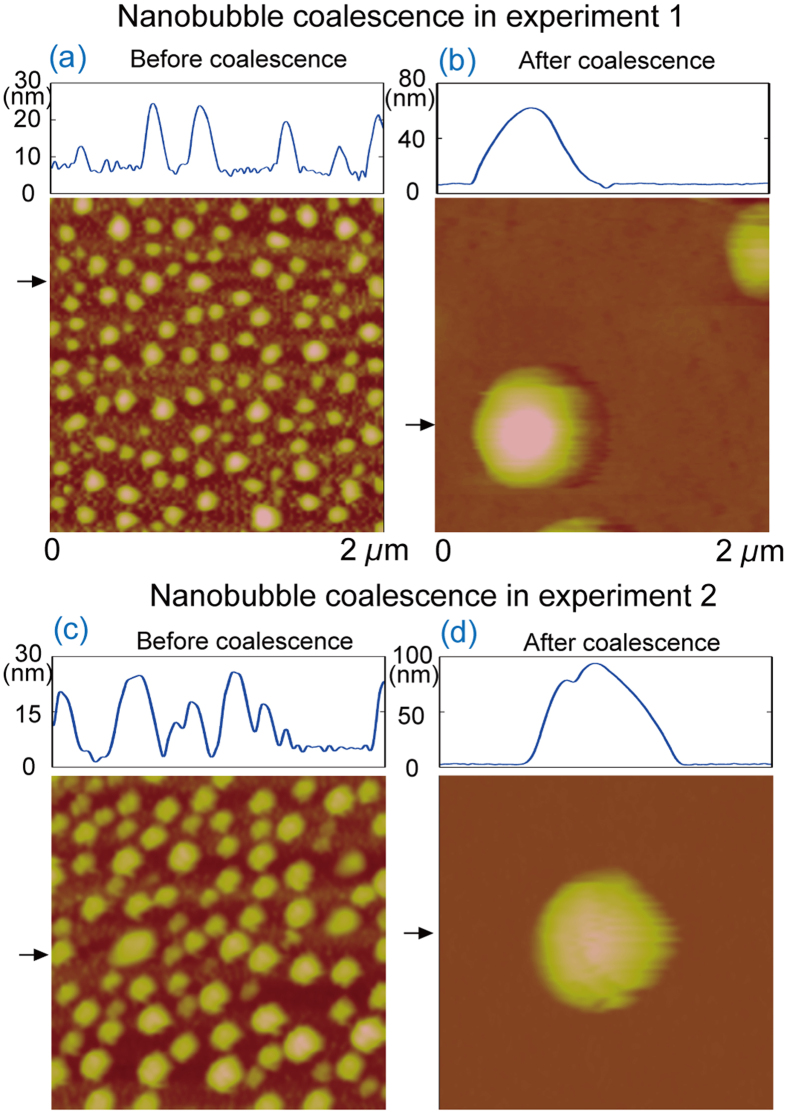
Nanobubble images before (**a**,**c**) and after coalescence (**b**,**d**) for experiment 1 and 2. After coalescence, nanobubbles with increased sizes were obtained.

After being immersed into deionized water (DI water), the polystyrene (PS) films were scanned using tapping mode AFM (TMAFM). The amplitude setpoint was 95% of the free oscillation amplitude of the cantilevers. The obtained AFM images for the two experiments are shown in Fig. 1a,c. In experiment 1 (Fig. 1a), nanobubbles are about 130 nm in diameter and 15 nm in height. In experiment 2 (Fig. 1c), the size of nanobubbles are slightly larger. The diameter is about 150 nm and the height is about 17 nm.

Nanobubble coalescence was conducted to increase the length of pyramidal interaction region. In this study, a higher scan load of 60% of free oscillation amplitude was applied to induce nanobubble coalescence^24^. The nanobubble images obtained after coalescence are shown in Fig. 1b,d for experiment 1 and experiment 2, respectively. For experiment 1, a nanobubble with 700 nm in diameter and 53 nm in height was obtained (Fig. 1b). For experiment 2, a slightly larger nanobubble of 850 nm in diameter and 95 nm in height was obtained (Fig. 1d).

### AFM Tip Geometry Construction

**Figure 2:**
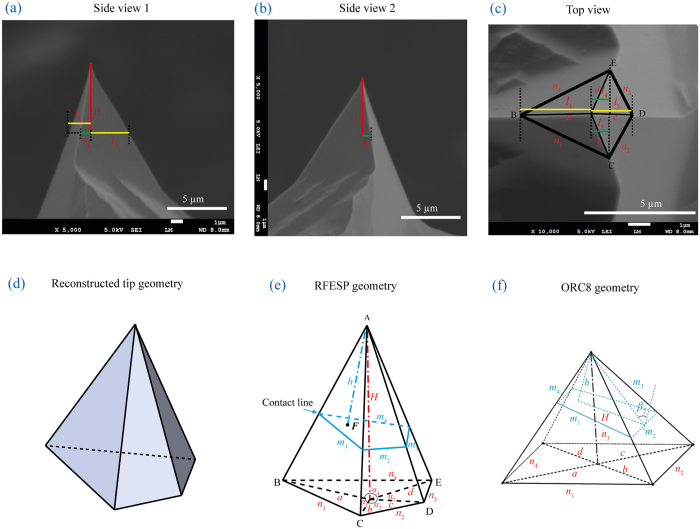
Geometry construction of AFM tips with SEM images. SEM images of side view 1 (**a**), side view 2 (**b**), and top view (**c**) for the RFESP tip. The tip geometry construction was performed through projection of some key dimensions among the different view images. (**d**) 3D geometry model for the constructed RFESP tip. (**e**) and (**f**) illustrate geometries and the perimeters of the three phase contact lines during tip-nanobubble interaction for RFESP and ORC8, respectively.

Most commercially available AFM cantilevers are silicon or silicon nitride (Si_3_N_4_). In this study, RFESP (silicon) and ORC8 (Si_3_N_4_) cantilevers were used. SEM imaging was performed on both types of tips using a field emission SEM (JSM-7500, JEOL, Japan). Tip geometry construction was achieved through projection of key dimensions among different images. This process is summarized visually in Fig. 2, taking the RFESP cantilever as an example.

The tip geometry construction is conducted as following. From the SEM image of Fig. 2a, a height *H* is first drawn and selected as a basic dimension. The horizontal distances *l*_1_, *l*_3_ and *l*_4_ from the three slide edges of the cantilever to the lower end of *H* were measured and projected to the top view SEM image (Fig. 2c). With *l*_1_, *l*_3_ and *l*_4_, the three intersection points B, C, and D on the base plane normal to *H* can be located. Similarly, the intersection point E can be located using *l*_2_ from Fig. 2b. After that, the horizontal distance from A to B, C, D, and E can be determined, denoted as *a*, *b*, *c*, and *d*. The angles between any two neighboring edges on the horizontal plane can also be determined, as *α*_1_, *α*_2_, *α*_3_, and *α*_4_ (Fig. 2e). With these dimensions, the tip can be geometrically constructed, as shown in Fig. 2d. The dimensions used in AFM tip geometry construction for the two cantilevers are listed in Table 1.

### Mathematic Model of Tip-nanobubble Interaction

To model tip-nanobubble interaction, two parameters need to be determined: the length of the three phase contact line and the angle of the side wall relative to the vertical direction. During scanning, there is an 11° incline between the long axis of the AFM cantilever and the horizontal plane. We define this plane as the working plane. In this paper, the perimeter of the intersection area between the constructed AFM tips and the working plane can be taken as three phase contact line. These are indicated in Fig. 2e,f for RFESP and ORC8 cantilevers, respectively. After the intersection is determined, the angle of the side wall on each side of the AFM tips can be determined. One example of the angle (*β*_*2*_) of the side wall is shown in Fig. 2f.

During indentation, there are two forces acting on the AFM tips. One is the force due to the pressure difference across the vapor-liquid interface^32^. The other is the capillary force along the three phase contact line. Therefore, the vertical force *F*_*vert*_ which can be detected by AFM can be given as:


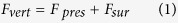


where *F*_*pres*_ and *F*_*sur*_ are vertical forces applied to AFM tips due to pressure difference and surface tension, respectively. Since nanobubbles are flat relative to the AFM tip during tip-nanobubble interaction, *F*_*pres*_ is much smaller than *F*_*sur*_. Therefore, only the capillary force is considered in the vertical direction. Moreover, we chose nanobubbles with large relative size where the volume of AFM tip inserted into the nanobubble will be much smaller than that of nanobubbles. Using the fact that nanobubbles appear flat to the tip, this means the displacement of the AFM scanner can be approximately taken as the indentation depth during tip-nanobubble interaction. For an indentation depth *h*(*z*), the vertical component of capillary force *F*_*sur*_ along the three phase contact line on four side walls of the AFM tips can be given as:


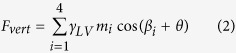


where *γ*_*LV*_ is the liquid vapor surface tension of water (72 mN m^−1^), *θ* is the contact angle, *m*_*i*_ is the length of the three phase contact line on the *i*th (*i* = 1, 2, 3, and 4) side wall of the tip, and *β*_*i*_ is the angle of the side wall relative to the vertical direction. In Eq. (2), the length of the three phase contact line *m*_*i*_ linearly increases with the indentation depth *h*(*z*) and is given as:


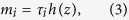


where *τ*_i_ is constant for *i*th side wall of the tip. By combining Eqs (2) and (3), the total vertical force can be given as:


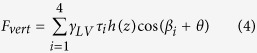


Since the tip end is spherical, the AFM tip cannot be taken as a perfect pyramid. Furthermore, during tip-nanobubble interaction, it is difficult to preciously determine the exact value of indentation depth. However, in the pyramidal interaction region, the surface tension length varies linearly with indentation depth; and the coefficient *τ*_*i*_ can be measured with much higher accuracy. In this study, we focus on the rate of change of the capillary force with respect to the indentation depth. Eq. (4) is then rewritten as:


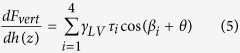


In Eq. (5), *θ*, *βi*, and *γ*_*LV*_ are constant. With the constructed cantilever geometries, *τ*_*i*_ and *β*_*i*_ for the two cantilevers can be obtained and are listed in Table 2.

Due to the existence of contact angle hysteresis, the vertical force applied to AFM tips during extension motion is different from that of the retraction motion. Taking the advancing and receding contact angles as *θ*_adv_ and *θ*_rec_, the derivatives of corresponding vertical forces 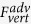 and 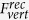 can be given as:


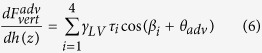


and


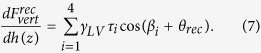


From Eq. (6) and Eq. (7), one can expect that during both extension and retraction motion of the AFM scanner, the capillary force should linearly increase with indentation depth. Since *θ*_*adv*_ is larger than *θ*_*rec*_, 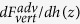 should be smaller than 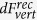
*/dh(z)*. Moreover, the dynamic contact angles *θ*_*adv*_ and *θ*_*rec*_ can be obtained by recording the capillary force during tip-nanobubble interaction.

### Detailed Process of Tip-nanobubble Interaction

**Figure 3:**
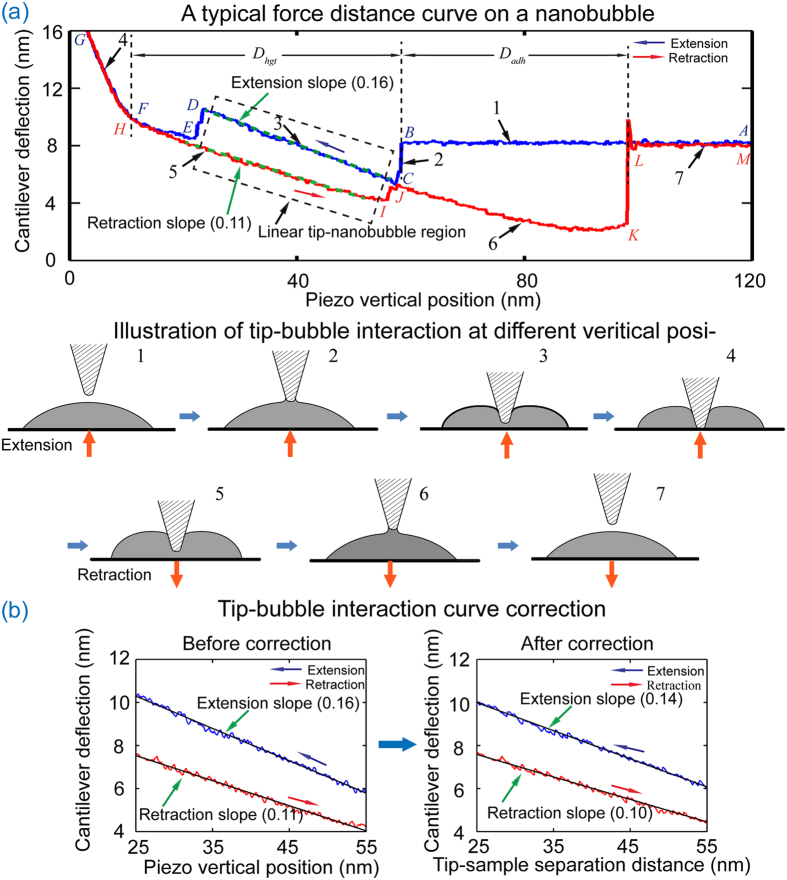
Tip-nanobubble interaction for the silicon nitride cantilever ORC8. (**a**) A typical force distance curve measured on a nanobubble. Insets illustrate the tip-nanobubble interaction along with the proceeding of the force-distance curve measurement at each section of 1–7 labeled in different stages, corresponding to features on the force distance curve. The force distance curve clearly shows the pyramidal interaction region. (**b**) Conversion of cantilever deflection–piezo position curves (left) to deflection–separation distance curves (right).

After coalescence, AFM probe tip insertion and removal was conducted on nanobubbles with each kind of AFM tip using force-volume mode measurement. The force distance curves obtained from testing the substrate are quite different from those obtained from nanobubbles. A typical force distance curve obtained on a nanobubble in experiment 1 with the ORC8 cantilever is shown in Fig. 3a. The whole tip-nanobubble interaction is divided into 7 sections. The insets shown in Fig. 3a illustrate the tip-nanobubble interaction at each section.

In the extension motion of the AFM scanner, the tip approaches the nanobubble. Before the tip gets contact with the nanobubble, there is no interaction force between them (inset 1 in Fig. 3a). This is reflected by the flat cantilever deflection signal in section 1 (points A→B). At point B, the AFM tip contacts the nanobubble, and a meniscus bridge forms between the tip and the nanobubble. The tip is rapidly drawn into the nanobubble due to the capillary force (inset 2 in Fig. 3a), as indicated by the sharp decrease in the cantilever deflection in section 2 (points B→C). At point C, the three phase contact line enters the pyramidal tip-nanobubble interaction region (inset 3 in Fig. 3a). As noted previously (see Eq. 3), in this section, the three phase contact line linearly increases with insertion depth of the cantilever tip into the nanobubble. Since the capillary force is linearly related to the length of the three phase contact line, the force should linearly increase with decreasing piezo vertical position. Linear force-deflection behavior is indeed observed in section 3 (points C→D). The obtained slope is about 0.16 in this section.

At point D, the cantilever tip approaches the sample surface. The tip is attracted to the sample surface due to electrostatic forces and Van der Waals forces. This is represented by a decrease in cantilever deflection with decreasing piezo vertical position. At point F, the tip contacts the solid sample surface (inset 4 in Fig. 3a). This hard contact dominates the cantilever deflection signal and results in a rapid linear increase of cantilever deflection with decreasing piezo vertical position in section 4 (F→G).

After the AFM scanner goes to the minimum vertical position, it reverses its motion and starts the retraction motion. From point G to point H, the tip-sample surface interaction force decreases with increasing piezo vertical position, resulting in a decreasing cantilever deflection signal (G→H). After that, the tip reenters the pyramidal tip-nanobubble interaction region, as shown in inset 5. In the corresponding section in Fig. 3a (H→I), the insertion depth of the AFM tip into the nanobubble linearly decreases with increasing piezo vertical position, which is behavior analogous to that in section 3. This is a result of decreasing length of the three phase contact line and decreasing interaction force. During retraction, the contact angle is an advancing contact angle. The observed slope of cantilever deflection v.s. piezo vertical position in section 5 is lower than that in section 3 of Fig. 3a, which is consistent with the derived mathematic model given in Eqs (6) and (7).

At point I, the three phase contact line retracts from the side walls of the tip, and the pyramidal interaction region ends. After the small transient between points I and J, the AFM tip enters the spherical contact region in section 6 (J→K). In this section, the three phase contact line is first pinned at the intersecting line of pyramidal and spherical interaction regions. With increasing retraction, the angle at the contact line between liquid-gas interface and vertical direction decreases, resulting in increased attraction force between the tip and the nanobubble (inset 6 in Fig. 3a). As retraction proceeds, the three phase contact line retreats from the boundary between the pyramidal and spherical regions, entering the spherical region. After that, the meniscus becomes thinner and thinner with increasing piezo vertical position, which results in decreasing tip-nanobubble interaction force. At the point *K*, the meniscus breaks and the cantilever deflection rapidly goes to zero in section 7 (L→M, inset 7 in Fig. 3a).

Several parameters can be extracted from Fig. 3a. The distance *D*_*hgt*_, the height of the nanobubble, is the vertical displacement the scanner travels from point B to point F (or H). During retraction, the tip does not lose contact with the nanobubble at the same displacement where it initially made contact. At point L, the meniscus bridge breaks and the AFM tip loses contact with the nanobubble. Therefore, the distance *D*_*adh*_, the vertical distance between points B and L, is related to the maximum adhesive force between the cantilever tip and the nanobubble.

In addition to *D*_*hgt*_ and *D*_*adh*_, the dynamic wetting properties can be investigated from the pyramidal interaction region (section 3 and section 5). In the two sections, the measured cantilever deflection signal as a function of piezo vertical position needs to be corrected to reflect the change of cantilever deflection with respect to tip-sample separation distance^2^. The left image in Fig. 3b is an enlarged plot of the area in Fig. 3a indicated by a dashed box. The linearly fitted slopes for extension and retraction motion are 0.16 and 0.11, respectively. The cantilever deflection signal was added with the piezo vertical position to get an accurate tip-sample separation distance. This is shown in the right half of Fig. 3b. The corrected slopes on the tip-sample separation distance plot are 0.14 and 0.10 for extension and retraction respectively.

**Figure 4:**
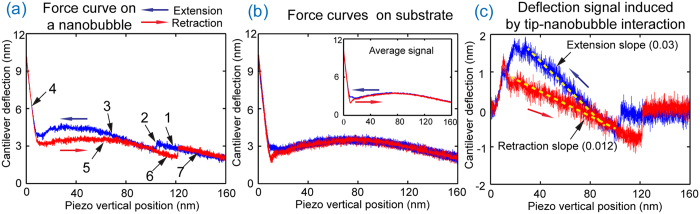
Tip-nanobubble interaction for the RFESP cantilevers. (**a**) A force distance curve obtained on a nanobubble. Stages of tip-nanobubble interaction are labeled in the same sequence as in Fig. 3 (**b**) Reference signal. Five superimposed force-distance curves for the sample substrate shown along with average signal. (**c**) Deflection signal of tip-nanobubble interaction obtained by subtracting the reference signal from experimental data. One can clearly see two slopes in extension and retraction motion, which correspond to the pyramid tip-nanobubble interaction region.

A typical force-distance curve obtained on a nanobubble in experiment 2 with the FRESP silicon cantilever is shown in Fig. 4a. Similar to that obtained with the ORC8 cantilever, the force curve was divided into seven sections. Since there was no gold coating on the back side of the RFESP cantilever, during experiment, the sum cantilever deflection signal from the AFM photo detector is low. It is also apparent from the curvature in sections 1 and 7 that optical interference^38^ has a large impact on the deflection signal. One can see that the force-distance curve is not flat even in section 1 and 7, where there is no tip-nanobubble interaction. Therefore, the cantilever deflection signal in tip-nanobubble sections shown in Fig. 4a has two components. One is the signal caused by tip-nanobubble interaction, the other is the signal caused by optical interference. In order to compensate for interference, a reference signal was used. The reference signal, shown in Fig. 4b, was the average of five force-distance curves from sample areas without nanobubbles^38^. Cantilever deflection curves for tip-nanobubble interaction, shown in Fig. 4c, were produced by subtracting the reference signal from raw experimental data.

One can clearly see two slopes in Fig. 4c, which correspond to extension and retraction motion of the AFM scanner during tip-nanobubble interaction. The slopes for approach and retraction are 0.03 and 0.012, respectively. The obtained curve is further corrected to get the accurate curve of cantilever deflection as function of tip-sample distance (data not shown). From the corrected curves, the slopes of 0.029 and 0.0119 are obtained for extension and retraction motions, respectively.

### Dynamic contact angle calculation

**Figure 5:**
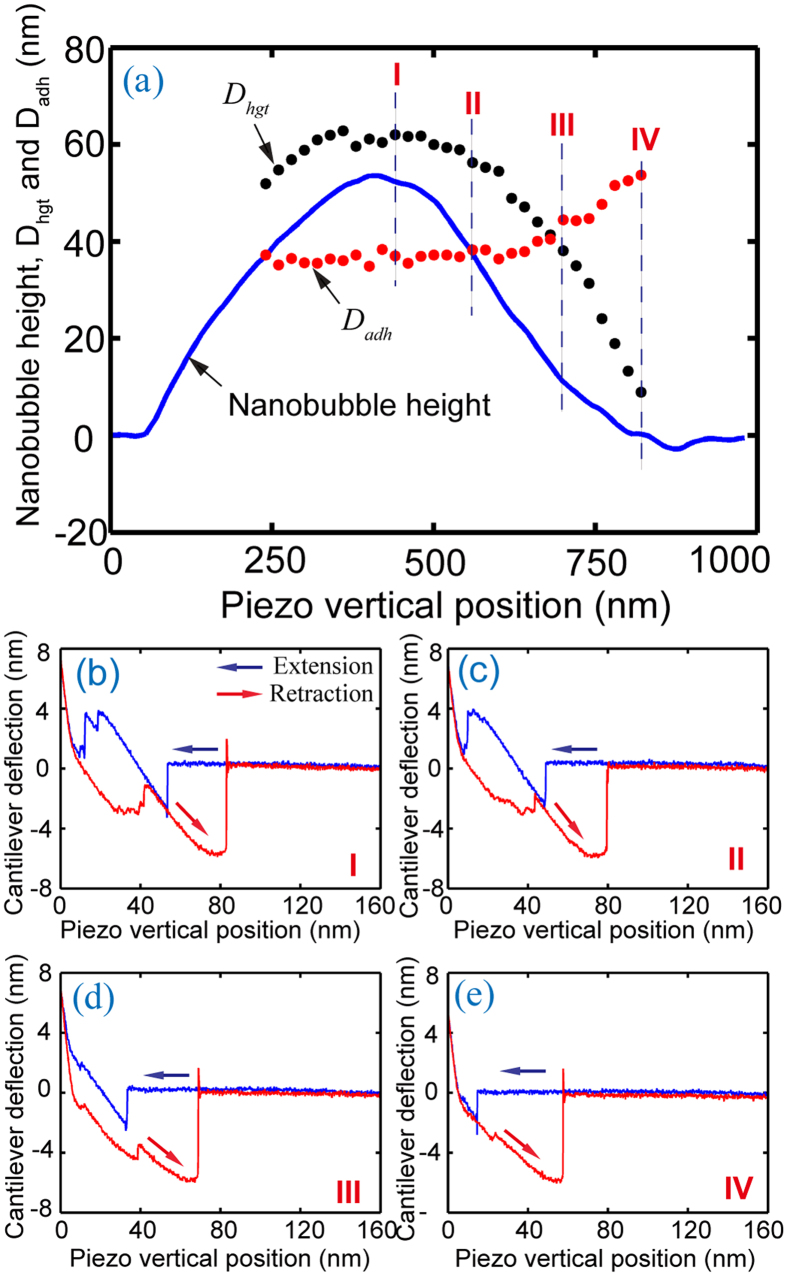
(**a**) *D*_*hgt*_ and *D*_*adh*_ obtained through force volume mode measurement and nanobubble cross section obtained through TMAFM scanning with the ORC8 cantilever. (**b–e**) Raw force-distance curves obtained from representative points, marked I, II, III, and IV in (**a**).

Force volume mode scanning was used in both experiments. In the force volume mode, the AFM cantilevers scan the area containing the nanobubbles with fixed step sizes along *x* and *y* axes. In the experiment 1, the scan area is 600 nm × 600 nm with step sizes of 20 nm in the *x* direction and 50 nm in the *y* direction. In the experiment 2, the scan area is 200 nm × 400 nm with step sizes of 10 nm and 50 nm along *x* and *y*, respectively. At each point, a force distance curve was obtained. For each force distance curve, the *D*_*hgt*_ and *D*_*adh*_ were manually identified. Figure 5 shows *D*_*hgt*_ and *D*_*adh*_ values for the nanobubble scanned with the ORC8 AFM tip along with representative force-distance curves.

In Fig. 5a, *D*_*hgt*_ and *D*_*adh*_ are plotted along a cross section of the nanobubble (shown in Fig. 1b). Four force distance curves from locations I, II, III, and IV are selected and shown in Fig. 5b–e. One can see that *D*_*hgt*_ first increases along the nanobubble cross section. After the maximum height, *D*_*hgt*_ decreases with decreasing nanobubble height, following the profile of the nanobubble. *D*_*hgt*_ is larger than nanobubble height. This is consistent with previous results^24^. The fact the *D*_*hgt*_ is larger than nanobubble height measured through TMAFM scanning implies that the TMAFM imaging underestimates nanobubble height^24^. Unlike *D*_*hgt*_, *D*_*adh*_ inversely change with nanobubble height. It has a low value at the nanobubble’s apex and achieves maximum value near the nanobubble’s boundary, as shown in Fig. 5a.

**Figure 6:**
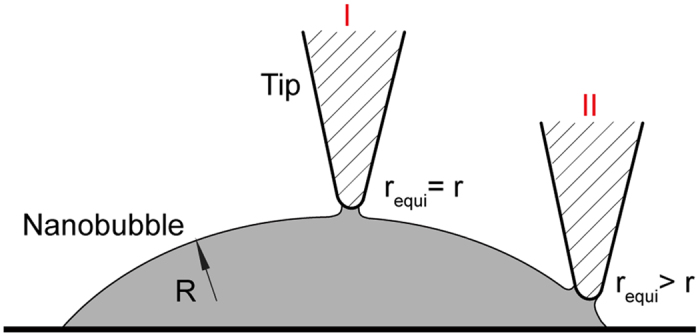
Illustration of the meniscus bridge formed between an AFM tip and a nanobubble. The contact area increases as the tip contacts the nanobubble at a more oblique angle toward the nanobubble’s edge, resulting in an increasing equivalent radius *r*_*equi*_.

The change of *D*_*adh*_ along nanobubble cross section is believed to be due to the change of contact area, as illustrated in Fig. 6. This change in contact area causes a corresponding change in adhesion force. The adhesive meniscus force *F*_*m*_ of a spherical tip in contact with the liquid on a planar surface can be given as^26^:


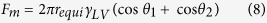


where *θ*_1_ and *θ*_2_ are the contact angle of the liquid with the tip and the plane surface, respectively, and *r*_*equi*_ is the equivalent radius of tip-nanobubble contact. Since the radius of the nanobubble is about 1000 times larger than that of the AFM tip, the nanobubble can be approximated as a planar surface when modelling the tip-nanobubble interaction. As a result, the angle *θ*_2_ is assumed to be zero and *F*_*m*_ can be taken as:


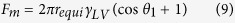


As shown in Fig. 6, the equivalent radius *r*_*equi*_ changes along the nanobubble cross section. At nanobubble apex (I in Fig. 6), *r*_*equi*_ is equal to tip radius *r* (*r*_*equi*_ = *r*). When the tip gets close to nanobubble boundary (II in Fig. 6), part of the side wall of the AFM tip comes in contact with the nanobubble, resulting in an increasing *r*_*equi*_ (*r*_*equi*_ > *r*). According to Eq. (9), the adhesive meniscus force *F*_m_ is proportional to *r*_*equi*_. Therefore, the adhesive force increases with decreasing nanobubble height.

**Figure 7:**
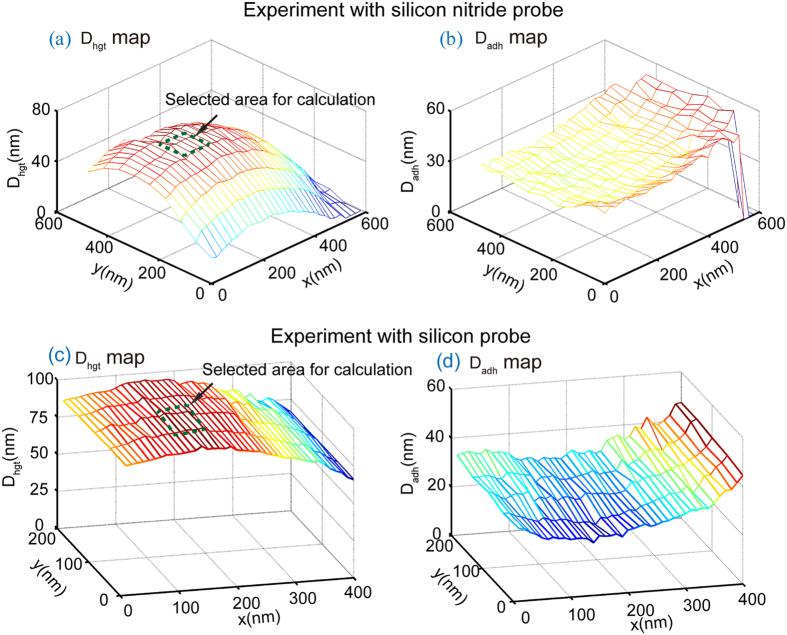
Constructed maps of *D*_*hgt*_ (**a**,**c**) and *D*_*adh*_ (**b**,**d**) for the experiment 1 (ORC 8 cantilever) and experiment 2 (RFESP cantilever), respectively.

Surface plots showing *D*_*hgt*_ and *D*_*adh*_ over the entire scan were constructed for each experiment and are shown in Fig. 7. Figure 7a,b show constructed maps of *D*_*hgt*_ and *D*_*adh*_ for the ORC8 probe, and Fig. 7c,d show constructed maps of *D*_*hgt*_ and *D*_*adh*_ for the RFESP probe. The change of *D*_*hgt*_ and *D*_*adh*_ over nanobubble surface is consistent with that shown in Fig. 5. *D*_*hgt*_ achieves the highest value at the apex of the nanobubbles and decreases towards the edge of the nanobubble, while *D*_*adh*_ has a minimum at the nanobubble’s apex and gradually increases towards the nanobubble’s edge.

With the force-distance curves over nanobubble surfaces, the dynamic contact angles for each AFM probe can be obtained using Eqs (6) and (7). Since the geometry of the three phase contact line varies at different locations on nanobubble surface (as indicated in Fig. 6), only the force distance curves obtained near the apex of the nanobubble were used for dynamic contact angle measurement. In this study, 60 nm × 50 nm scan areas around the nanobubble apex (green dashed box in Fig. 7a,c) were selected for dynamic contact angle calculation. The mean values of slope for the force distance curves in the selected areas were calculated for extension and retraction; these are listed in Table 3.

With the slopes in the pyramidal interaction region, the dynamic contact angles can be calculated using Eqs (6) and (7). The advancing contact angle *θ*_*adv*_ and receding contact angle *θ*_*rec*_ for the silicon cantilever (RFESP) are 61.8 ± 1.6° and 44.3 ± 0.7°, respectively. This is larger than the values reported for bulk silicon samples, 56.6° and 42.1° 39. For the silicon nitride cantilever (ORC8), the calculated values of *θ*_*adv*_ and *θ*_*rec*_ are 46.3 ± 1.1° and 42.0 ± 0.7°, respectively. Dynamic contact angles are seldom reported for silicon nitride. The reported static contact angle (*θ*_s_) of bulk silicon nitride samples is 32° 40. Given the relationship *θ*_adv_ > *θ*_s_ > *θ*_rec_, the contact angles measured here are larger than expected in comparison to reported values for both materials.

The difference between these results and reported values for bulk materials may be due to line tension along the three phase contact line^41^,^42^ or due to differing surface heterogeneity between macroscopic and microscopic samples^19^,^43^. At macroscale, based on the classical Young’s equation, the contact angle can be given as:


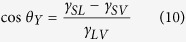


where *θ*_*Y*_ is the static contact angle at macroscale, *γ*_*SL*_ and *γ*_*SV*_ are the solid-liquid and solid-vapor interfacial tensions, respectively. At nanoscale, the effect of line tension at the three phase contact line on contact angles becomes larger and is not negligible. The classical Young’s equation needs to be modified as:


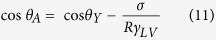


where *θ*_*A*_ is the actual static contact angle, *σ* is the line tension along the three phase contact line, *R* is the radius of the three phase contact line. The contact angles in Eqs (10) and (11) are static ones. Here the average values of the advancing and receding contact angles are taken as the static contact angles. The static contact angles for silicon and silicon nitride cantilevers at nanoscale are 53.0° and 44.2°, respectively. By substituting the static contact angles into Eq. (11) and approximately taking the three phase contact line in the pyramidal interaction region as a circle with perimeter of about 150 nm (*R* ≈ 24 nm), the obtained line tensions are 1.0 × 10^−10^ N and 2.2 × 10^−10^ N for the experiments with the silicon and the silicon nitride cantilevers, respectively. The values are closed to that of 0.7 × 10^−10^ N reported by Pompe and Herminghaus on silicon surface^42^.

The proposed method provides a direct measurement of dynamic contact angles. The method could be beneficial for the fundamental understanding of the dynamic wetting properties of nanostructures or in AFM tip related applications. However, from Eqs (6) and (7), one can see that the accuracy of the dynamic contact angle measurement is directly related to that of the measured AFM tip geometries, *τ*_*i*_ and *β*_*i*_. The two parameters were obtained through tip geometry reconstruction from SEM images of the AFM tips. The AFM cantilevers are normally soft and the vibration amplitude caused by thermal noise is relative high. This limits the resolution of SEM images when selected magnifications were high during imaging. The limited SEM image resolution reduces the measurement accuracy of *τ*_*i*_ and *β*_*i*_, which is believed to be the main source of errors for the dynamic contact angle measurements.

## Conclusion

In this study, the dynamic wetting properties of silicon and silicon nitride AFM tips were investigated at the nanoscale using a nano-Wilhelmy based approach. Nanobubbles were used to provide stable liquid-vapor interfaces with minimized signal fluctuation. Geometric models were constructed for the both AFM tips using SEM images taken from different viewing angles. The entire tip-nanobubble interaction was divided into two sections: a spherical interaction region and a pyramidal interaction region. A mathematic model which includes parameters of dynamic contact angles was derived for the pyramidal interaction region.

Using force distance curves obtained from force volume mode scans of nanobubbles, the detailed process of AFM tip-nanobubble interaction was investigated. *D*_*hgt*_, the height of the nanobubble, and *D*_*adh*_, the parameters direction related to the maximum adhesion distance between the tip and nanobubble, were extracted from each force-distance curve. Experimental results showed that the measured *D*_*hgt*_ increased with increasing nanobubble height and reached a maximum value at the nanobubble’s apex. Conversely, *D*_*adh*_ decreased with increasing nanobubble height, having a low value at the nanobubble apex and reaching a maximum value at nanobubble boundaries. The change in *D*_*adh*_ is ascribed to the change in effective contact area between the AFM tip and the nanobubble at different locations over the nanobubble surfaces. Toward the edges of the nanobubbles, there is a larger effective contact area than on the nanobubble apex.

In the force distance curves, the snap-in and snap-out sections correspond to interaction with the spherical region of the AFM tip. The two sections where the cantilever deflection signal changes linearly with tip-sample separation distance correspond to the pyramidal interaction region. In this region, the slope of the capillary force with respect to the separation distance is larger for extension than for retraction; this is consistent with the mathematic model developed herein and implies the existence of contact angle hysteresis. Using force-distance data from the nanobubble apexes, the advancing *θ*_*adv*_ and receding contact angle *θ*_*rec*_ were calculated to be 66.2 ± 1.6° and 55.6 ± 0.7° for silicon and 46.3 ± 1.1° and 42.0 ± 0.7° for silicon nitride AFM tips. These values are larger than the values reported for bulk materials. There are two plausible causes for this discrepancy: the existence of line tension along the three phase contact line, or the difference in surface heterogeneity between macroscopic and microscopic contact angles. To the best of our knowledge, this is the first time the dynamic contact angles of AFM tips were directly measured. Moreover, this work provides an approach for investigation of micro/nanoscale dynamic wetting property measurement, which is crucial for applications in bioassays, micro-reactors, and chemical and biological sensing.

## Methods

### Principle of Dynamic Contact Angle Measurement

**Figure 8:**
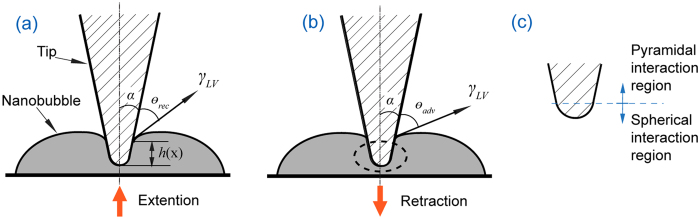
Schematic of AFM tip-nanobubble interaction during extension (**a**) and retraction (**b**) of the AFM scanner. Based on tip geometry, the interaction is divided into two regions, spherical interaction region and pyramidal interaction region (**c**).

In this study, the dynamic contact angles are extracted through AFM tip-nanobubble interaction. This interaction during extension and retraction of the AFM scanner is illustrated in Fig. 8. In the figure, *α* is the angle between the side wall of the AFM tip and vertical direction, *θ*_*rec*_ and *θ*_*adv*_ are receding and advancing contact angles of the AFM tip, respectively, γ_*LV*_ is the liquid vapor surface tension, and *h*(*x*) is the depth of indentation into the nanobubbles.

From a geometrical point of view, the AFM tip can be divided into two regions, the spherical region at the end of the AFM tip and the upper pyramidal region. This is shown schematically in Fig. 8c. In the spherical region, the free end of AFM tip can be regarded as spherical in shape. In the upper pyramid region, the tip is pyramidal in shape, and its cross section linearly increases along tip height direction. Accordingly, the tip-nanobubble interaction can be divided into two corresponding regions: a spherical interaction region and a pyramidal interaction region. In the spherical interaction region, precise measurement of the detailed geometry is difficult. Furthermore, tip-nanobubble interaction in this region is complex. It is not clear if the meniscus will interact only with in the spherical portion of the AFM tip or if it will advance directly to the intersection between the spherical region and the pyramid part of the AFM tip^12^,^44^.

In the pyramidal interaction region, the perimeter of the three phase contact line linearly increases with indentation depth *h*(*x*). The tip geometry can be measured with relatively high accuracy through SEM imaging. Therefore, the angle *α* can be measured, and the perimeter of the three phase contact line can be obtained for a given *h*(*x*). In this study, the dynamic contact angles *θ*_rec_ and *θ*_adv_ are obtained by measuring the force on the AFM tips during extension and retraction of the AFM scanner.

### Nanobubble Imaging and Coalescence

In this study, two experiments (1 and 2) were conducted with the Si_3_N_4_ cantilever ORC8 (Bruker, USA) and the silicon cantilever RFESP (Bruker, USA), respectively. For each experiment, a polystyrene (PS) sample was prepared by spin-coating thin film of PS on a silicon (100) substrate at a speed of 500 rpm. Before spin-coating, the Silicon (100) substrates were cleaned in a sonication bath of acetone and then water. PS particles (molecular weight 350,000, Sigma-Aldrich) were dissolved in toluene (Mallinckrodt Chemical) to a concentration of 0.2% (weight). The contact angle of the PS surfaces with water was measured using a sessile drop method to be 95 ± 3°. During experiment, samples were immersed in DI water.

In this study, a commercial AFM (MultiMode III, Digital Instruments) operating in tapping mode was used for nanobubble imaging and coalescence in DI water. To obtain an improved signal-to-noise ratio, a modified tapping mode tip holder was applied, as in previous work^24^,^45^. The resonance frequencies of the cantilevers in water were measured to be 26.5 KHz and 18.2 KHz for the RFESP and the ORC8 cantilevers, respectively. A drive frequency close to each cantilever’s resonance frequency was used during imaging. A scan rate of 2 Hz with a 90° scan angle and a 2 μm × 2 μm scan area was used for both imaging and coalescence. The cantilever stiffness was calibrated via thermal noise method^46^. The calibrated stiffness was 3.3 ± 0.3 N/m and 0.73 ± 0.07 N/m for RFESP and ORC8 cantilevers, respectively.

Nanobubbles have merit because they provide stable liquid-gas interfaces for study. However, the nanobubbles that spontaneously form on PS surfaces are too small to be used for dynamic wetting property investigation in this experiment. To increase the tip insertion depth during tip-nanobubble interaction, nanobubbles with diameters around 400 nm were obtained by performing nanobubble coalescence^24^.

During nanobubble imaging, the setpoint was set to be only 95% of the free oscillation amplitude to minimize the force applied on nanobubbles and sample surface. To induce coalescence, higher scanning loads were used at 60% of the free oscillation amplitude. After nanobubble coalescence, 95% setpoint scanning was performed again to verify that the nanobubbles had reached an acceptable size.

### Force Volume Measurement of Tip-nanobubble Interaction

Force volume mode operation was used during scans to get series of force-distance curves for the AFM tip wetting property investigation. In force volume mode, the AFM scanner performs a vertical extension and retraction relative to the specimen surface at different positions along *x* and *y*; and curves relating force as a function of distance are obtained for each scan point. During indentation, the cantilever oscillation voltage is switched off. To reduce the influence of hydrodynamic forces on the measurement results^2^, a low vertical extension and retraction speed was used. The ramp size for the extension and retraction motion is 200 nm and the scan rate is about 0.5 Hz. This corresponds to a 200 nm/s extension or retraction speed. In this experiment, different step sizes were chosen along *x* and *y* axes. Step sizes of 10 nm in *x* and 50 nm in *y* were used in the experiment with the RFESP cantilever. For the experiment with the ORC8 cantilever, the step sizes of 20 nm in *x* and 50 nm in *y* were used. The smaller step sizes along the *x* axis give a high spatial resolution of scan points over the nanobubble surfaces, whereas the larger step sizes along the *y* axis reduce the overall scan time.

In force volume mode operation, it is common practice to use trigger voltages of cantilever deflection. During extension of the AFM tip, the cantilever deflection signal increases with increasing tip-sample interaction. When the deflection signal reaches a predetermined trigger voltage, the AFM scanner stops and begins to retract. In this study, the trigger voltage function was not used; instead, extension and retraction was limited to a selected vertical range. This guarantees that the vertical position where AFM tips contact the sample surface is consistent and facilitates the alignment of force curves obtained from different locations. As a result, the nanobubble topography can be constructed with the force-distance curves obtained in force volume operation.

## Additional Information

**How to cite this article**: Wang, Y. *et al*. Nano-Wilhelmy investigation of dynamic wetting properties of AFM tips through tip-nanobubble interaction. *Sci. Rep*. **6**, 30021; doi: 10.1038/srep30021 (2016).

## Figures and Tables

**Table 1:** Key dimensions obtained through dimension projection among different view images for tip geometry construction.

Cantilever	*H*(*μ*m)	*a*(*μ*m)	*b*(*μ*m)	*c*(*μ*m)	*d*(*μ*m)	*α*_1_(*°*)	*α*_2_(*°*)	*α*_3_(*°*)	*α*_4_(*°*)
RFESP	5.09 ± 0.10	3.20 ± 0.05	2.17 ± 0.04	1.87 ± 0.04	2.16 ± 0.04	114.26 ± 1.71	68.47 ± 1.30	66.89 ± 1.20	110.38 ± 1.87
ORC8	3.75 ± 0.06	3.12 ± 0.06	3.10 ± 0.06	3.55 ± 0.07	3.45 ± 0.06	92.5 ± 1.67	90.7 ± 1.54	84.7 ± 1.52	92.06 ± 1.66

**Table 2:** Parameters *τ*_i_ and *β*_i_ obtained from the constructed AFM tip geometries.

Cantilever	*β*_1_ (*°*)	*β*_2_ (*°*)	*β*_3_ (*°*)	*β*_4_ (*°*)	*τ*_1_	*τ*_2_	*τ*_3_	*τ*_4_
RFESP	9.96 ± 0.20	27.68 ± 0.50	27.68 ± 0.50	11.03 ± 0.20	0.87 ± 0.01	0.48 ± 0.01	0.49 ± 0.01	0.89 ± 0.01
ORC8	29.3 ± 0.48	42.8 ± 0.73	33.9 ± 0.61	20.3 ± 0.30	1.27 ± 0.02	1.47 ± 0.02	1.35 ± 0.02	1.15 ± 0.02

**Table 3:** Dynamic contact angles for experiments obtained with the RFESP and ORC8 cantilevers.

Cantilever	Extension slope	Retraction slope	*θ*_adv_ (°)	*θ*_rec_ (°)
Silicon (RFESP)	0.029 ± 0.001	0.012 ± 0.001	61.8 ± 2.1	44.3 ± 0.7
Silicon Nitride (ORC8)	0.139 ± 0.006	0.102 ± 0.010	46.3 ± 1.1	42.0 ± 0.7
